# Assessing the Agonistic Continuum Scale as a Measure of Sexual Sadism in a Sample of Community Members and BDSM Practitioners

**DOI:** 10.5964/sotrap.13829

**Published:** 2024-09-10

**Authors:** Myles Davidson, Jay Healey

**Affiliations:** 1 Department of Psychology, Carleton University, Ottawa, ON, Canada; 2 Department of Criminology, Saint Mary’s University, Halifax, NS, Canada; Saint Mary's University, Halifax, NS, Canada

**Keywords:** sexual sadism, Agonistic Continuum, The Agonistic Continuum Scale

## Abstract

Sexual sadism refers to a sexual preference for fantasies and behaviours involving the infliction of humiliation, degradation, and suffering onto others. The conceptualization of sexual sadism remains a contentious issue in the literature, with some believing sexual sadism is qualitatively distinct from other deviant sexual preferences, while others suggest it lies on a continuum of sexually aggressive behaviours. This second approach, known as the Agonistic Continuum, is a relatively novel conceptualization of sexual sadism. Its companion scale, The Agonistic Continuum Scale (TACS), was created to measure sexual sadism as a dimensional construct in both forensic and community samples. Despite several validation studies being conducted, the factor structure of the TACS has yet to be independently assessed. As such, the current study sought to assess the factorial validity and measurement invariance of the TACS in a community sample made up of primarily BDSM practitioners using a series of confirmatory factor analyses and latent profile analyses. 248 Canadian adults (65.3% females, 75.6% BDSM practitioners) completed a survey containing a demographic questionnaire and several measures of paraphilic interest including the TACS. Results of the factor analyses suggested a four-factor model provided the best fit to the data. However, incorporating sex as a multigroup analysis factor rendered this model a significantly worse fit. Further, latent profile analysis results supported the ability of the TACS to identify groups differing in sexual sadism severity but returned significantly different fit statistics across sex groups. Taken together, while the results of past studies were partially replicated, our findings call into question the appropriateness of the TACS in mixed-sex and non-community samples.

## Highlights

- The links between sexual sadism and paraphilic coercion remain debated in the literature.
- The four-factor Agonistic Continuum solution provided the best fit to the data.
- The results highlight the importance of considering sample makeup when using the TACS.

Sexual sadism was first described by von Krafft-Ebing (1886) as the sexual arousal toward behaviours and fantasies relating to the infliction of pain and suffering onto others. Research since this initial description has attempted to identify the key components of sexual sadism. In addition to the synergy between sex and aggression, some of the core features of sexual sadism are believed to be the exertion of power and control, the infliction of pain, humiliation, degradation, bondage, and fantasies related to these behaviours (Alison et al., 2001; Knight et al., 1994; Langevin, 1983; Marshall & Kennedy, 2003; Yates et al., 2008). Sexual sadism has also long been considered to play a role in sexual homicide offences, though the extent of this role remains debated (Geberth & Turco, 1997; Langevin et al., 1988; Marshall & Kennedy, 2003; Meloy, 2000). Since its initial conceptualization, sexual sadism has been a difficult construct to both operationalize and reliably measure due to disagreements as to what behavioural and cognitive elements are necessary for its identification (Marshall & Kennedy, 2003). This, in turn, has led to the emergence of various competing perspectives on sexual sadism as a construct. In the following paper, we will discuss a more recent conceptualization of sexual sadism, as well as our efforts to replicate existing research on its companion measure.

Despite elements of sexual coercion being present in sexually sadistic behaviour, arousal toward sexual coercion and sexual violence have long been considered representative of distinct interests. The more recent revelation of potential links existing between the arousal toward sexual violence (sexual sadism) and the arousal toward forced sex acts (paraphilic coercion or biastophilia), however, has introduced a new debate to the literature. Historically, paraphilic coercion was considered part of the courtship disorders and represented an anomaly arising during the sexual intercourse stage of courtship (Freund & Blanchard, 1986; Freund & Seto, 1998). As such, it was considered distinct from sexual sadism which was seen as part of the algolagnic (literally meaning lust for pain) disorders (Freund & Seto, 1998). This distinction has long been supported by phallometric data demonstrating differences in the arousal patterns of perpetrators of rape and those with an interest in sexual sadism. Specifically, perpetrators of rape tend to demonstrate a sexual preference for non-consenting sexual encounters, whereas those with an interest in sexual sadism tend to demonstrate a sexual preference for sexually violent behaviour (Harris et al., 2012; Seto et al., 2012). More recently, an opposing position has suggested that the arousal patterns of these two groups are more similar than distinct, suggesting that paraphilic coercion may itself be a component of sexual sadism (Knight et al., 2013; Longpré et al., 2020).

This new position, referred to as the Agonistic Continuum (agonistic referring to the infliction of agony onto others), describes a continuum encompassing sexual fantasies and behaviours related to bondage, paraphilic coercion, and severe sexual sadism (D’Orazio & Flinton, 2021; Knight et al., 2013; Longpré et al., 2020). In its current form, the Agonistic Continuum places non-coercive sexual fantasies and behaviours at the lower end of the continuum, followed by fantasies and behaviours related to the practice of BDSM (bondage, discipline, domination, submission, and sadomasochism; Longpré et al., 2020). Paraphilic coercion, or fantasy and behaviours related to non-consent, is situated at the center of the continuum, with severe sexual sadism (i.e., torture, mutilation, sexual homicide) being situated at the upper end (Longpré et al., 2020). Past research examining the Agonistic Continuum has generally provided support for the dimensional structure of sexual sadism (Knight et al., 2013; Longpré et al., 2018, 2019, 2020).

Having established this new explanation of sexual sadism, Knight et al. (2013) proposed the Agonistic Scale. The Agonistic Scale is a 17-item measure designed to measure fantasies and behaviours related to the Agonistic Continuum. Using factor analysis, Item Response Theory, and taxometric analysis, Knight et al. (2013) determined that sexual sadism and paraphilic coercion were highly overlapping constructs that were better explained as existing along a single dimension rather than being distinct factors. These findings were later replicated by Longpré et al. (2020) who added that the presence of an additional general factor further supported the notion that sexual sadism is part of an underlying dimension of sexually coercive behaviour. Research by Thornton (2019), although disagreeing with respect to the number of factors captured by the Agonistic Scale, also provided support for the existence of a general factor. To address some of the limitations of the Agonistic Scale, Longpré et al. (2019) proposed the modified Agonistic Continuum Scale (TACS). This new 30-item scale conceptualizes sexual sadism as a higher-order unidimensional construct comprised of four interrelated factors, each corresponding to a different section of the Agonistic Continuum: Coercion and Power (paraphilic coercion), Bondage/Humiliation (BDSM), Physical Violence/Beating (severe sexual sadism), and Killing (severe sexual sadism). Initial taxometric analyses using this scale have further supported the dimensional nature of sexual sadism, and Item Response Theory analyses have suggested the scale is a valid measure of the Agonistic Continuum in both community and forensic samples (Longpré et al., 2019).

## The Present Study

While the TACS appears to be a valid measure of the Agonistic Continuum, further research into this scale is needed. Aside from the work of the scale contributors, no independent validation studies on the TACS have been carried out. Replication of past findings is necessary to determine whether the scale is a truly viable measure of sexual sadism in different types of samples. As the TACS has previously been validated for use in both forensic and community samples, the purpose of this study was to assess the factor structure of the TACS in a novel sample made up of primarily BDSM practitioners. Furthermore, while past studies (e.g., Longpré et al., 2019) have measured sexual sadism using the TACS in mixed samples containing males and females, sex differences between males and females with respect to the factor structure of the scale have not been explored. Therefore, this study also sought to explore whether the TACS is appropriate to use with both males and females by measuring its fit to the data when using sex as a multigroup factor. As the goal of this study was to determine whether past findings examining the TACS could be replicated, we hypothesized that our model fit indices would resemble those reported in past studies using this scale.

## Method

### Sample

The sample for this study was collected as part of the first author’s master’s thesis. This thesis study sought to assess the relationship between involvement with an organized BDSM community, an interest in sexual sadism, and experience with consent violations. In total, *N* = 248 Canadian adults were recruited from three distinct groups. The first group (*n* = 60, 24.2%) were members of the general population who had no interest in BDSM and did not practice BDSM regularly. The second group (*n* = 125, 50.4%) were those possessing at least a moderate interest in BDSM and who practiced BDSM regularly. The third group (*n* = 63, 25.4%) were those possessing at least a moderate interest in BDSM and who practiced BDSM regularly within the context of an organized BDSM community. As we were interested only in examining males and females, the two participants who did not disclose their biological sex were excluded from the sample, resulting in a final sample size of *N* = 246. This sample was considered satisfactorily large for factor analysis as it exceeded the recommended minimum sample size of *N* = 200 (Hoe, 2008). Most of the sample were self-reported BDSM practitioners (*n* = 186, 75.6%), and almost two-thirds were female (*n* = 162, 65.9%). For additional demographic information, see Table 1.

**Table 1:** Demographic Characteristics of the Study Sample

Variable	*M*	*SD*
**Age**	28.53	7.72
**Number of sexual partners**	8.27	14.19
Variable	*N*	%
Gender		
Woman	152	61.8
Man	74	30.1
Non-binary	12	4.9
Transgender	1	0.4
Other	7	2.8
Sexual orientation		
Straight	170	69.1
Mostly straight	16	6.5
Bisexual	28	11.4
Mostly gay/lesbian	6	2.4
Gay/lesbian	9	3.7
Pansexual	9	3.7
Asexual	2	0.8
Other	6	2.4
Ethnicity^a^		
North American origins	156	63.4
European origins	74	30.1
Caribbean origins	8	3.3
Latinx	2	0.8
Central and South American origins	2	0.8
African origins	7	2.8
Asian origins	12	4.9
Oceanian origins	1	0.4
Other	2	0.8
Prefer not to say	1	0.4
Experience with sadistic activities^b^		
Never tried	52	21.4
Tried once or twice	86	35.4
Try regularly	105	43.2
Sexual interest in sadistic activities^b^		
Sadistic acts are repulsive	108	44.4
Indifferent	92	37.9
Sadistic acts are arousing	43	17.7

*Note*. *N* = 246.

^a^Participants could select more than one ethnicity. ^b^Experience with and sexual interest in sadistic activities were measured using the Paraphilias Scale (Seto et al., 2012).

### Measures

#### The Agonistic Continuum Scale (TACS)

The TACS (Longpré et al., 2019) was developed to measure sexual sadism as a dimensional construct. It contains 30 items across four subscales that are meant to capture the four facets of sexual sadism: Coercion and Power (e.g., “I have thought about forcing someone to have sex”), Bondage/Humiliation (e.g., “I have humiliated someone during sex”), Physical Violence (e.g., “I have purposely hurt someone physically during sex”), and Killing (e.g., “I have strangled someone during sex”). All items are measured on a five-point scale (0 = never, 1 = once, 2 = sometimes, 3 = fairly often, 4 = very often) and are tallied across each subscale, with higher scores on any given subscale denoting a stronger presence of that feature of sexual sadism. Scores on the TACS can range from 0 to 120, with maximum scores on each subscale being 32, 24, 24, and 40 on the Coercion and Power, Bondage/Humiliation, Physical Violence/Beating, and Killing subscales, respectively. The TACS has previously been validated by Longpré et al. (2019) on three different samples comprised of community members, university students, and non-sexual offenders where interitem correlations were identified in the moderate-to-strong range (*r* = .64 – .77). Past research has also demonstrated that the TACS is positively and moderately-to-strongly correlated with other measures of sexual sadism and sexual aggression (e.g., Paraphilias Scale, Sadomasochism Checklist, Sexual Experiences Survey; Davidson, 2023; Koss et al., 2006; Seto et al., 2012; Weierstall & Giebel, 2017).

### Procedure

This study received clearance from the ethics review board at Saint Mary’s University (REB #22-083). Recruitment of participants for this study took place across two waves. First, the study was advertised using social media [e.g., Twitter (X)] and recruitment posters posted at five sex education shops throughout Halifax, Nova Scotia, as well as on campus at Saint Mary’s University. Second, participants with an interest in BDSM were recruited from eight forums on FetLife and three forums on Reddit, as well as through making direct contact with several BDSM clubs across Canada, two of whom shared the recruitment materials with their members. Most participants were recruited through social media and online forums (*n* = 219, 88.3%). To be eligible to participate, prospective participants had to be at least 18 years of age, had to reside in Canada at the time of the study, and had to have access to an electronic device capable of accessing the Internet.

Prospective participants were invited to complete a survey on Qualtrics and were first greeted with an informed consent form that highlighted the purpose of the study and what information would be gathered from them. Those who consented to participating in the study were then able to complete the survey. Participants began the survey by answering a series of demographic questions, followed by measures of paraphilic interest, the TACS, and two measures of consent violation victimization and perpetration. Following the completion of the survey, participants interested in receiving compensation were redirected to a separate survey where they provided their email address to enter a draw for one-of-five $100 CAD Amazon gift cards.

### Data Analysis

A series of confirmatory factor analyses (CFA) were used to assess the factor structure of the TACS. Past research has suggested that the Agonistic Continuum is a higher-order unidimensional construct comprised of four interrelated latent factors (Knight et al., 2013, 2018; Longpré et al., 2018, 2019, 2020; Thornton, 2019). The TACS, proposed by Longpré et al. (2019), uses each of its four subscales to represent the four latent factors. In addition to this conceptualization, Thornton (2019) proposed an alternative conceptualization of the Agonistic Continuum which he saw as being comprised of two factors: Brutality (the Physical Violence/Beating and Killing subscales of the TACS) and Paraphilic Coercion (the Coercion and Power and Bondage/Humiliation subscales of the TACS). Furthermore, both Thornton (2019) and Longpré et al. (2020) suggested the presence of an additional general factor onto which all items would load, and which would support the theory that sexual sadism is part of an underlying dimension of sexually coercive behaviour. To assess each of the above conceptualizations, five main CFA models were created using the TACS (described below). In addition to these main models, to determine whether there were differences in model fit across participant sex, an additional multigroup CFA was computed using the best fitting model by including sex as a multigroup analysis factor. To account for missing data, full information maximum likelihood (FIML) estimation was used. All CFAs were conducted using SPSS AMOS. Additionally, a series of latent profile analyses (LPA) were conducted to determine whether scores on the TACS could be grouped into one or more latent classes. This was an attempt to replicate Longpré et al. (2020)’s study of the original Agonistic Scale demonstrating that, should it exist along an Agonistic Continuum, sexual sadism can be grouped according to behaviours that differ in terms of severity and not kind. All LPAs were conducted using Jamovi.

## Results

### TACS Descriptives

Internal consistency for the TACS in this study was very strong for the total scale (McDonald’s ω = 0.94). Internal consistency was acceptable for the Coercion and Power (McDonald’s ω = 0.77) and Bondage/Humiliation (McDonald’s ω = 0.79) subscales, and good for the Physical Violence/Beating (McDonald’s ω = 0.82) and Killing (McDonald’s ω = 0.89) subscales. The mean TACS score for the sample was 1.42 (*SD* = 0.85), with considerable range in responses (0 – 101). In general, scores were highest on the Coercion and Power subscale (*M* = 1.81, *SD* = 1.06), followed by the Bondage/Humiliation (*M* = 1.61, *SD* = 1.00), Physical Violence/Beating (*M* = 1.40, *SD* = 1.06), and Killing (*M* = 1.02, *SD* = 0.78) subscales. In this sample, interitem correlations ranged from weak to strong (*r* = .09 – .64). The means and ranges for all scale items can be found in Table 2.

**Table 2:** Descriptive Statistics for the TACS

Item	*M*	*SD*	Min	Max
Coercion and Power Subscale				
I have fantasized about dominating someone sexually	2.30	1.62	0	4
Making someone do what I want turns me on sexually	2.21	1.67	0	4
It turns me on to think about overpowering someone sexually	2.22	1.76	0	4
I have thought about forcing someone to have sex	1.67	1.62	0	4
I have fantasized about sexually abusing someone who is drunk or high on drugs	1.44	1.59	0	4
I have attempted sexual intercourse with someone who was drunk or high on drugs	1.84	1.79	0	4
I have had sex with someone who didn’t want to have sex with me	1.40	1.63	0	4
I have threatened to use physical force to make someone go along with sex	1.38	1.67	0	4
Bondage/Humiliation Subscale				
I have had sexual thoughts about tying my partner to a bed, legs and arms spread apart	1.85	1.35	0	4
While having sex, I have used handcuffs, whips, or leathers	1.88	1.42	0	4
I have tied someone up while we were having sex	1.63	1.40	0	4
I have humiliated others to keep them in line	1.39	1.43	0	4
I have thought about embarrassing or humiliating someone during sex	1.42	1.41	0	4
I have humiliated someone during sex	1.49	1.43	0	4
Physical Violence/Beating Subscale				
I have thought about threatening or frightening someone	1.39	1.39	0	4
I have daydreamed about how good it would feel to hurt someone during sex	1.43	1.44	0	4
I have hurt people for my own enjoyment	1.43	1.43	0	4
The more scared a person becomes, the more sexually turned on I get	1.39	1.55	0	4
I have purposely hurt someone physically during sex	1.41	1.51	0	4
While having sex, I have enjoyed scaring my companion so that they begged me to stop	1.38	1.51	0	4
Killing Subscale				
I have thought about choking someone during sex	1.20	1.13	0	3
I have thought about torturing someone during sex	1.12	1.11	0	3
I have thought about cutting someone with a knife	0.95	1.09	0	3
I have beaten someone while I was having sex	1.08	1.12	0	3
I have strangled someone during sex	1.13	1.15	0	3
I have thought about burning someone during sex	0.96	1.07	0	3
I have thought about killing someone during sex	0.93	1.10	0	3
I have fantasized about killing someone during sex	1.00	1.15	0	3
I enjoy seeing other people getting killed	0.93	1.13	0	3
I have tortured animals	0.86	1.03	0	3

*Note.* *N* = 246.

Table 3 contains the means and standard deviations of each subscale and the total scale separated by sex (male vs female) and BDSM interest (practitioner vs non-practitioner). Tests for differences in these means across sex groups demonstrated that females scored significantly higher than males on the Physical Violence/Beating subscale, *t*(235) = 2.69, *p* = .01, the Killing subscale, *t*(234) = 2.62, *p* = .01, and the total scale, *t*(226) = 2.40, *p* = .02. Additionally, BDSM practitioners scored significantly higher than non-BDSM practitioners on the Coercion and Power subscale, *t*(236) = 4.42, *p* < .001, the Bondage/Humiliation subscale, *t*(238) = 4.71, *p* < .001, and the total scale, *t*(226) = 3.40, *p* < .001.

**Table 3:** Descriptive Statistics for the TACS Separated by Sex and BDSM Interest

Group	Coercion and Power		Bondage/ Humiliation		Physical Violence/ Beating		Killing		Total	
	*M*	*SD*	*M*	*SD*	*M*	*SD*	*M*	*SD*	*M*	*SD*
Sex										
Male	1.70	1.14	1.53	1.11	1.15	1.06	0.83	0.86	1.24	0.92
Female	1.87	1.02	1.63	0.93	1.54	1.05	1.11	0.73	1.52	0.79
BDSM interest										
Practitioner	1.98	1.00	1.78	0.96	1.47	1.05	1.06	0.76	1.53	0.79
Non-practitioner	1.30	1.11	1.10	0.93	1.17	1.09	0.87	0.83	1.10	0.92

*Note*. *N* = 246.

### CFA Results

A series of CFAs were conducted to explore whether the factor structure of the Agonistic Scale identified in past research could be replicated using the TACS. Prior to analysis, multivariate normality was assessed for all 30 indicator variables where it was determined that the assumption was not violated. In total, five main models were assessed. These models were previously assessed by Longpré et al. (2020) using the Agonistic Scale and included the following: (a) a one-factor baseline model, (b) the two-factor model proposed by Thornton (2019), (c) the four-factor model proposed by Knight et al. (2013), (d) the bifactor model containing the two factors proposed by Thornton (2019) and a general factor, and (e) the bifactor model containing the four factors proposed by Knight et al. (2013) and a general factor.

**Figure 1:**
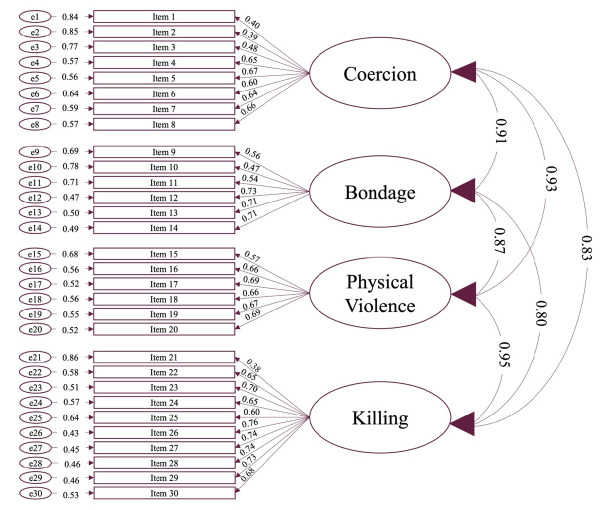
Path Model for the Four-Factor Agonistic Continuum Solution

Table 4 depicts the fit statistics for each CFA model. The one-, two-, and four-factor models were less than adequate fits to the data, with both the CFI and TLI falling below their acceptable thresholds in all three models. Calculation of chi-square difference tests suggested there was a significant improvement in fit between the one- and two-factor models, χ^2^(1, *n* = 246) = 81.00, *p* < .001, and the two- and four-factor models, χ^2^(5, *n* = 246) = 35.00, *p* < .001. That said, the RMSEA for the one- and two-factor models fell within the 90% confidence interval for the four-factor model, suggesting comparable fit across all three models. In each of the above models, all indicators loaded onto their respective factors, and all factor loadings were above the generally accepted threshold of .30. In contrast to the first three models, the two- and four-factor bifactor models were both good fits to the data, with the CFI and TLI both falling above their acceptable thresholds in these models. Calculation of chi-square difference tests suggested there was a significant improvement in fit between the four-factor model and the two-factor bifactor model, χ^2^(27, *n* = 246) = 233.00, *p* < .001, as well as between the two-factor bifactor model and the four-factor bifactor model, χ^2^(7, *n* = 246) = 58.00, *p* < .001. Additionally, the RMSEA for the two-factor bifactor model did not fall within the 90% confidence interval for the four-factor bifactor model, suggesting a significant difference in fit across the models. Despite the fit indices being favourable, the bifactor models returned anomalous results in that the presence of a general factor resulted in several negative factor loadings and abnormal variance estimates, suggesting the data may not have been appropriate for using bifactor models (Eid et al., 2017). As such, despite the less than adequate fit, the four-factor model was retained for further exploratory analyses. The path diagram for the four-factor model can be found in Figure 1.

**Table 4:** CFA Fit Indices for Several TACS Models

Model	χ^2^	*df*	RMSEA	90% CI	SRMR	CFI	TLI
One-factor	992.08***	405	.08	[.07, .08]	.07	.82	.81
Two-factor	910.68***	404	.07	[.07, .08]	.07	.84	.83
Four-factor	875.53***	399	.07	[.06, .08]	.07	.85	.84
Bifactor 2	643.44***	372	.06	[.05, .06]	.04	.92	.90
Bifactor 4	584.65***	365	.05	[.04, .06]	.04	.93	.92
Four-factor multigroup	1626.13***	798	.09	[.09, .10]	.08	.78	.76

*Note*. *N* = 246.

***p < .001.

Since the four-factor model had the best fit overall, it was chosen for the assessment of measurement invariance. A multigroup CFA was computed using the four-factor model with the inclusion of sex as the multigroup analysis factor. Table 4 presents the results of this model. This multigroup model was a significantly worse fit to the data than the four-factor model, with both the CFI and TLI falling well below their acceptable thresholds. Based on the poor fit of this model, configural invariance could not be established, and further invariance testing was not carried out.

### LPA Results

In their study on the Agonistic Scale, Longpré et al. (2020) determined that the Agonistic Scale could identify two distinct classes of individuals who were distinguishable according to sadistic behaviours that differed in severity and not in kind, providing support for the Agonistic Continuum. To determine whether scores on the TACS could also be grouped into classes representing different levels of severity, a series of LPA analyses were conducted. In line with Longpré et al. (2020), the indicator variables used were the subscale means for all four TACS subscales. Table 5 depicts the results of the LPAs. In total, six models were tested representing 1-, 2-, 3-, and 4-class solutions, as well as two models representing 2-class solutions for males and females separately. The Bayesian information criterion (BIC) indices decreased at each step, and both the Lo-Mendel-Rubin (LMR) likelihood statistic and bootstrapped likelihood ratio test (BLRT) were significant at each step (*p* < .05). Though this suggested explanatory power could be increased by adding additional profiles, due to a lack of theoretical rationale, minimal improvements to the BIC between the 2- and 3-class solutions, and significant overlap between profiles in the 3- and 4-class solutions, the two-profile solution was considered the preferred model.^1^1It should also be noted that the addition of further profiles beyond the 2-class solution did not change the interpretation of the findings generated by the 2-class solution.

**Table 5:** Latent Profile Analysis Fit Statistics

Model	LL	AIC	BIC	LMR *p*	Classification Accuracy
1-class	-1262	2541	2568	–	–
2-class	-942	1911	1955	< .001	.99 – .99
3-class	-927	1889	1951	< .001	.74 – .99
4-class	-857	1760	1839	< .001	.88 – .99
2-class males only	-355	735	766	< .001	.98 – .99
2-class females only	-564	1154	1193	< .001	.99 – .99

*Note.* LL = log-likelihood; AIC = Akaike information criterion; BIC = Bayesian information criterion; LMR *p* = *p*-value for the Lo-Mendel-Rubin likelihood test.

Table 6 presents the latent profile mean estimates for this 2-class solution. Examination of the 2-class solution suggested that the classes differed in the severity of sexual sadism exhibited rather than in kind. In this 2-class solution, 61.4% of cases were assigned to the high-severity class, with the remaining 38.6% being assigned to the low-severity class. The high-severity class was marked by significantly higher scores on all subscales than the low-severity class, with the largest differences being seen for both the Physical Violence/Beating and Killing subscales. Additionally, an examination of the 2-class solutions for males and females separately revealed that the 2-class solution for just males had significantly stronger fit indices than the 2-class solution for just females. Otherwise, the pattern of findings for both males and females mirrored that of the 2-class solution for the entire sample.

**Table 6:** Latent Profile Mean Estimates for the Two-Class TACS Solution

TACS Subscale	Latent Profile Mean Estimates			
	Low-Severity		High-Severity	
	*M*	*SE*	*M*	*SE*
Coercion and Power	0.81	0.71	2.43	0.70
Bondage/Humiliation	0.72	0.70	2.18	0.69
Physical Violence/Beating	0.25	0.42	2.11	0.66
Killing	0.18	0.23	1.54	0.47

*Note*. *SE* = standard error.

## Discussion

The purpose of this study was to attempt to replicate past research on the Agonistic Scale by examining the factor structure of the TACS in a novel sample, as well as to determine whether this factor structure is applicable to both males and females. In partial support of our hypotheses, we were able to successfully replicate some of the findings of previous validation studies (e.g., Longpré et al., 2020) by demonstrating that the TACS appears to capture the four-factor structure of the Agonistic Continuum originally proposed by the Agonistic Scale, despite each of the models tested having a less than ideal fit. Additionally, the LPA analyses mostly supported the findings of Longpré et al. (2020) and demonstrated that, like the Agonistic Scale, scores on the TACS can be used to meaningfully categorize individuals according to the severity of their sadistic behaviour rather than the type of behaviour they exhibit. Despite these general consistencies between our findings and those we attempted to replicate, we highlight new concerns relating to the use of the TACS, specifically as it relates to the composition of samples for which it is appropriate to be used.

The CFA results in this study are mostly in line with those reported by Longpré et al. (2020) and demonstrate that the four-factor structure of the Agonistic Continuum, originally established using the Agonistic Scale, can be replicated using the TACS, albeit with a less than ideal fit. We add to the literature by demonstrating that this four-factor structure appears to be visible in a sample made up primarily of BDSM practitioners, although the fit indices fall below the generally accepted thresholds. While our results also provide preliminary support that the addition of a general factor onto which all items load leads to a significantly better fit to the data, our bifactor models produced anomalous results due to data limitations. As such, while the findings at face value provide some support for the presence of a general factor, future studies replicating Longpré et al. (2020)’s bifactor models are needed to properly assess the factor structure of the TACS. Furthermore, while this study was able to shed additional light on the factor structure of the TACS in the context of past studies (e.g., Knight et al., 2013; Longpré et al., 2019, 2020), a full validation of the TACS, including the degree to which it captures the dimensionality of sexual sadism proposed by the Agonistic Continuum, was beyond the scope of this study. Given that the TACS is a novel measure of sexual sadism, future studies should strive to carry out full validations of the TACS to better assess its construct validity, its convergent and discriminant validity with other measures, and its ability to capture the proposed dimensionality of sexual sadism.

This study is unique in that the number of females in the sample provided the opportunity for measurement invariance testing based on sex. Despite historically being used in mixed-sex samples (Longpré et al., 2019), we were unable to establish configural invariance between sexes in this study. Specifically, our findings demonstrated that the four-factor model with sex as a multigroup analysis factor was a significantly worse fit to the data than the standard four-factor model. This finding lends itself to two potential explanations. First, it is possible that sexual sadism as a construct may be noninvariant, meaning it manifests differently in males and females. Given the significant TACS subscale mean differences observed between males and females in this sample, it is possible that the TACS is capturing something unique about sexual sadism across the sexes. Preliminary research suggests there may be some distinct characteristics of sexual sadism in females as compared to males (e.g., psychological torture with nurturing components; Pflugradt & Allen, 2012); however, given the lack of research on sexual sadism in females, future research is needed to test this non-invariance hypothesis. Second, this finding may be due to the overrepresentation of females in our sample and not because of a true difference. Past research has identified that severely unbalanced group sizes may lead to incorrect conclusions about invariance (Yoon & Lai, 2018). Nonetheless, our study provides preliminary evidence that the TACS may not be appropriate for use with mixed-sex samples.

Another finding of note related to the TACS and sex is that females in our sample scored significantly higher than males on two of the TACS subscales, as well as the total scale. As mentioned above, this unexpected finding may be related to the overrepresentation of females in our sample and may not be a true difference. Relatedly, this finding may be due to the fact that, because there were more females than males in this sample, the proportion of female BDSM practitioners was notably larger than the proportion of male BDSM practitioners. Alternatively, as mentioned earlier, the TACS may be capturing something unique about sexual sadism in females that is leading to these higher subscale scores. Future research is needed to determine whether this observed difference is stable across similar samples or simply a unique feature of this sample.

The results of the LPA analyses mirror those of Longpré et al. (2020) in that scores on the TACS, much like the original Agonistic Scale, can be used to create two groups of individuals whose behaviour differs in severity and not kind. Specifically, those in the high-severity group scored higher on all four TACS subscales than those in the low-severity group, especially the Physical Violence/Beating and Killing subscales. Despite the above-reported invariance findings, we chose to carry out the LPA analyses across sexes to determine whether they would shed additional light onto the multigroup CFA findings. These LPAs for males and females separately demonstrated that the model fit was significantly better for males than females. While further research is needed to explore the nuances of this finding, it provides additional support for the non-invariance finding and further highlights the need to revisit the TACS and determine whether it is indeed appropriate to use the scale with mixed-sex samples.

An additional finding of note from the 2-class LPA solution is that two-thirds of participants in this sample were classified as “high severity”, meaning they tended to score higher on the TACS items than those in the “low severity” class. These findings are in direct contrast to Longpré et al. (2020) who found that only 7.7% of their sample were high severity. This difference is likely explained by the number of BDSM practitioners in this sample, especially considering the group differences on the TACS subscales we identified between BDSM practitioners and non-BDSM practitioners. While it is believed that 8-10 percent of those in the general population have BDSM-related interests (Brown et al., 2020), over half of our sample self-disclosed possessing these interests. As such, the unique composition of this sample may have contributed to some of the divergent findings, meaning it is imperative that this scale be assessed in other community samples that are more representative of the general population. That said, the large number of self-identified BDSM practitioners in this sample provided the unique opportunity to explore which types of samples the TACS can be used with in its current state. Since the TACS was a less than ideal fit to the data in this sample, it is unclear whether it is appropriate for use in samples where the prevalence of sexual sadism interest is higher than average. Given our sample size limitations, however, future studies should seek to replicate these findings to determine whether model fit improves in a larger sample with a similar prevalence of sexual sadism interest. Additionally, future studies should seek to determine whether measurement invariance can be established between BDSM and non-BDSM practitioners. As this study was only interested in assessing the TACS in the context of this novel sample, and due to the limited number of non-BDSM practitioners in the sample, measurement invariance testing using this multigroup factor was beyond the scope of this study.

In summary, this study highlights some of the strengths of the TACS as a measure of the Agonistic Continuum while simultaneously highlighting areas for further improvement. As this is the first known study to independently assess the factor structure and measurement invariance of the TACS, future studies must carry out full validations of the TACS in addition to attempting to replicate the present findings while addressing the limitations. Furthermore, there is a need for additional research determining whether this scale is appropriate to be used with mixed-sex samples and with community samples where the prevalence of sexual sadism is higher than average (e.g., BDSM communities). Nonetheless, while our study was only able to provide preliminary findings on the factor structure of the TACS, we were able to demonstrate that the composition of the sample should be a principal consideration when deciding when to use a scale designed to measure sexual sadism, especially as it relates to the sex of those completing the measure.

### Limitations

This study is not without limitations. First and foremost, the sample size for this study was smaller than what is typically seen in studies using factor analysis and latent profile analysis. While there is no one rule of thumb, it is generally thought that one should have a sample size of a least *N* = 200 to carry out these analyses (Hoe, 2008), though some rules suggest a sample size of at least *N* = 300 is necessary (Tabachnick & Fidell, 2001), suggesting that the present study may have been slightly underpowered. Additionally, given that neither sex group contained more than *n* = 200 participants, the multigroup CFA and LPAs were also likely slightly underpowered. As such, all findings reported herein should be interpreted with some caution, and future studies should attempt to replicate these findings in larger samples. That said, we believe the uniqueness of this sample and the fact that this study serves a starting point for validation research with the TACS outweighs any potential sample size limitations. Second, our sample was comprised of significantly more females than males. While this afforded the unique opportunity to examine how the TACS performed with a sample made up primarily of females, it is possible that this overrepresentation impacted the findings. Third, while the four-factor CFA model was a less than ideal fit to the data, we proceeded with the remaining analyses for the purpose of exploration. As such, all results should be interpreted with some caution. Lastly, a taxometric analysis using the TACS, as well as an assessment of its convergent and discriminant validity, was beyond the scope of the present study. As mentioned earlier, the novelty of this scale necessitates further independent validations.

## Data Availability

The data used in this study are available from the corresponding author upon reasonable request.

## Social Media Accounts

@MylesDavidson98

## Related Versions

This study was derived from exploratory analyses conducted on the TACS as part of the first author’s master’s thesis.
